# Influence of Crimped Steel Fibre on Properties of Concrete Based on an Aggregate Mix of Waste and Natural Aggregates

**DOI:** 10.3390/ma13081906

**Published:** 2020-04-17

**Authors:** Jacek Katzer, Janusz Kobaka, Tomasz Ponikiewski

**Affiliations:** 1 Faculty of Geoengineering, University of Warmia and Mazury in Olsztyn, 10-720 Olsztyn, Poland; 2 Faculty of Civil Engineering, Environmental and Geodetic Sciences, Koszalin University of Technology, 75-453 Koszalin, Poland; janusz.kobaka@tu.koszalin.pl; 3 Faculty of Civil Engineering, Silesian University of Technology, 44-100 Gliwice, Poland; tomasz.ponikiewski@polsl.pl

**Keywords:** aggregate, white ceramic, red ceramic, waste, fibre, SFRC

## Abstract

This research was inspired by the growing global shortage of natural aggregates. Different types of waste ceramics (apart from recycled concrete) are the most popular materials for the production of waste aggregates as possible substitutes for natural ones. The aim of this research was to analyse the efficiency of different aggregate mixes composed of waste and natural materials focusing on two waste ceramic aggregates, which were prepared concrete mixes based on specifically composed aggregates (blend of natural aggregate, porous and iron oxide-rich (red) waste ceramic aggregate, and dense, kaolin-based (white) waste ceramic aggregate). All aggregates were thoroughly tested before utilisation for concrete mix creation. Altogether, four blends of aggregates were prepared in order to prepare concrete mixes using a simplex experiment design. The mixes were then modified by adding various amounts of crimped steel fibre. Such properties of hardened steel fibre-reinforced concrete (SFRC) such as density, compressive strength, shear strength, ultrasound propagation velocity, dynamic modulus of elasticity, and limit of proportionality during flexural testing were of special interest. Tests were conducted according to European and Japanese standards. The achieved fibre-reinforced concretes were characterised by satisfactory strength characteristics, thereby enabling the substitution of traditional reinforcement. Strength classes according to the *fib* Model Code 2010 were assigned.

## 1. Introduction

A growing research effort exists globally to successfully harness different ceramic wastes in the construction industry [1,2,3], resulting in some successful applications of different types of waste ceramics as partial full substitutes of fine and coarse natural aggregates [4,5,6]. The type of waste ceramic most often considered for harnessing as a waste aggregate is red (porous, iron oxide-rich) ceramic [4,7], with multiple types of nonstructural concrete elements characterised by less demanding strength characteristics being cast using this kind of waste aggregate [8]. In order to utilise waste ceramic aggregates for the production of structural concrete, a new approach to composition is needed. Different waste ceramic aggregates could be blended together (e.g., red waste ceramic aggregate and white (dense, kaolin-based) waste ceramic aggregate) to achieve a new level of quality in sustainable concrete production, thereby enabling the shaping of aggregate properties to utilise their advantages and harness synergy. Red ceramic is characterised by limited compressive strength due to its porosity (usually between 10 and 15 MPa), but has the advantage of using an internal curing process [7]. White ceramic is characterised by “no porosity” and a much higher compressive strength red ceramic. This research was conducted to prove the proposed novel concept of using waste ceramic aggregates, in which red ceramic obtained from brick production waste and white ceramic obtained from local pottery factory production waste were used as waste aggregates. Both ceramics were processed using the same machinery and grinding procedure to achieve waste aggregates. As a reference, the properties of natural post-glacial aggregates commonly available in countries located along the southern shoreline of the Baltic Sea [9] were chosen. Utilisation of a mixture design was enabled using three aggregates, where the sum of the volume of all three ingredients was always equal to 100%. The mixture design allowed the visualisation of results in the form of ternary contour plots, which are commonly used in technology of binders [10] and concrete [11].

The research programme was divided into two stages. The first stage covered the testing of the geometrical and mechanical properties of the waste and natural aggregates. The second stage covered property testing of concretes made on the basis of the waste and natural aggregates that were tested during the first stage. Analysis of the possible replacement of natural aggregates by waste ceramic aggregates was subsequently conducted. Specific mixtures of both waste ceramic and natural aggregates were proposed for concrete production. The obtained four mixes were subsequently modified by the addition of steel fibres, which were added in volumes ranging from 0.5% to 1.5% (*V_f_*). Altogether, 16 mixes of concrete were cast in order to test the properties of the concretes in a hardened state.

## 2. Materials

**Figure 1:**
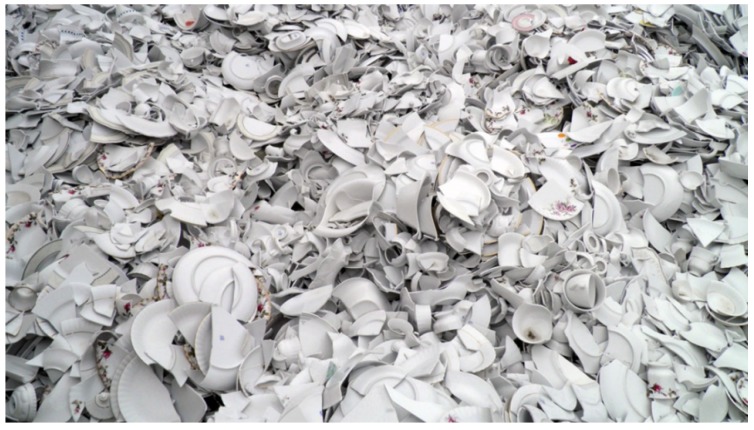
White ceramic waste sourced from a local pottery producer.

**Figure 2:**
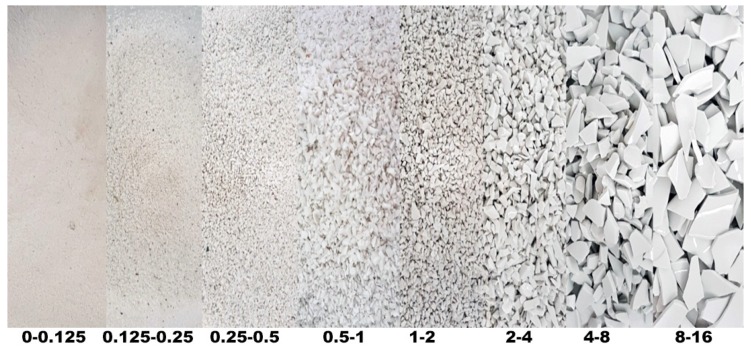
Fractions of white ceramic waste aggregate.

The aggregates were specially prepared for the research programme. Both raw ceramic wastes were ground into particle fractions from 0 to 16 mm. White ceramic waste in a form of crushed pottery rejected during production was used for waste aggregate preparation, as seen in Figure 1. The aggregate particle fractions and particle geometry achieved from this process are shown in Figure 2. The particles were characterised by sharp edges with some rough and smooth surfaces. The shape of particles was also very different from the shape of the sub-rounded grains of the natural aggregate.

**Figure 3:**
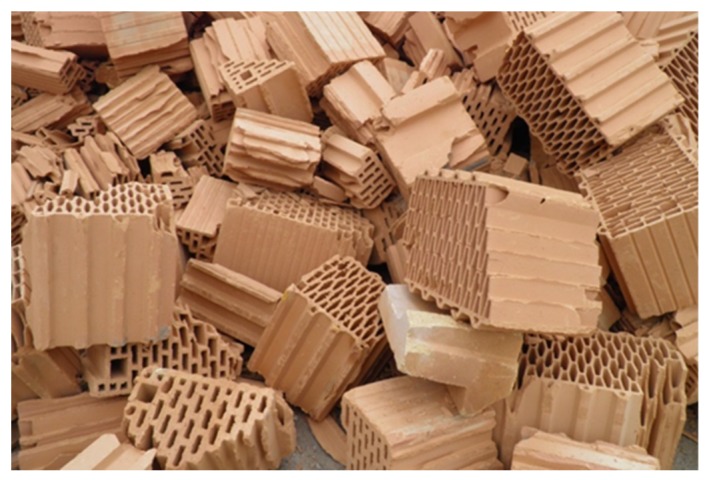
Red ceramic waste sourced from a local brick producer.

**Figure 4:**
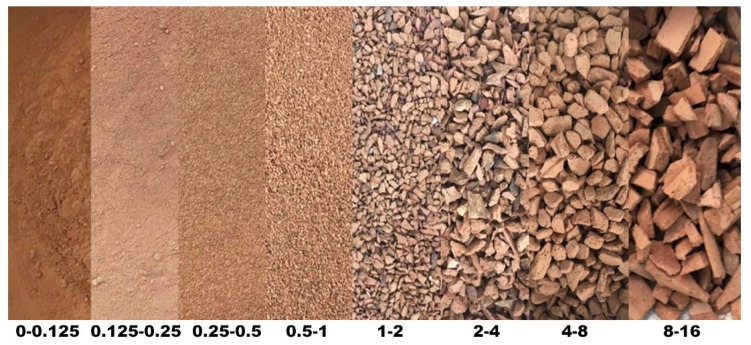
Fractions of red ceramic waste aggregate.

Sourced red waste ceramic is presented in Figure 3. It mainly comprised hollow ceramic wall blocks either crushed during production or rejected during final quality control. Aggregate particle fractions and their geometric characteristics are shown in Figure 4. This aggregate was thoroughly described in previous publications [12].

**Figure 5:**
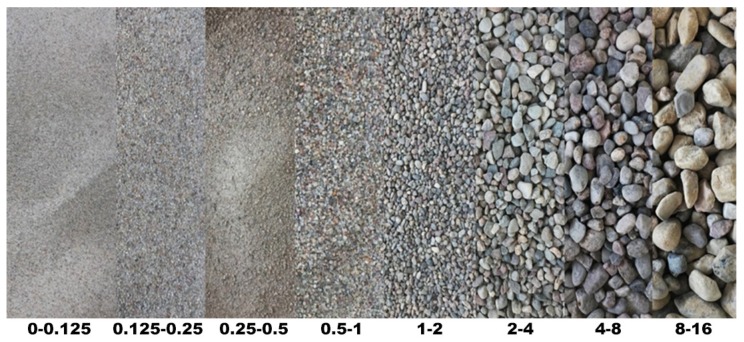
Fractions of natural post-glacial aggregate.

The natural aggregate of post-glacial origin used as a raw material for the creation of the third kind of aggregate used in this research is shown in Figure 5. This aggregate was thoroughly described in numerous previous publications [9,13,14].

All three aggregates were sieved into separate fractions. The fractions were composed to fulfil the limits of the grading characteristics of fine aggregate used for concrete and cement testing, as described in European (EN 196-1:2016) and American (ASTM C305) standards. The achieved grading of all three aggregates was very similar (see Table 1). All three composed aggregates were characterised by a median diameter *d_m_* [15] within the range from 3.33 to 3.67 mm. 

The aggregates were thoroughly tested according to the European standards EN 933-1, EN 1097-1,-3,-6, and EN 1367-1. Properties such as density, absorbability, freezing–thawing resistance, and abrasion resistance were of special interest. The Los Angeles abrasion test was utilised as the most common test method to indicate aggregate toughness and abrasion characteristics. Granulometric and other tested properties of the aggregates are presented in Table 1.

Portland Cement CEM I 42.5 N-NA compliant with the EN 197-1:2000 standard was used as a binder for concrete mix creation. Tap water meeting the requirements of the EN 1008:2002 standard and high-performance, commercially available superplasticiser were added during the last stage of the mix preparation. Based on previous experience with the aggregates in question [16], four different aggregate compositions were created (see experimental design). Cast concrete mixes were characterised by w/c = 0.5 and an aggregate/cement ratio of 3.0. These characteristics mirrored the requirements for cement composite mixes used for tests of cement compressive strength, according to EN 196-1 standard. 

**Figure 6:**
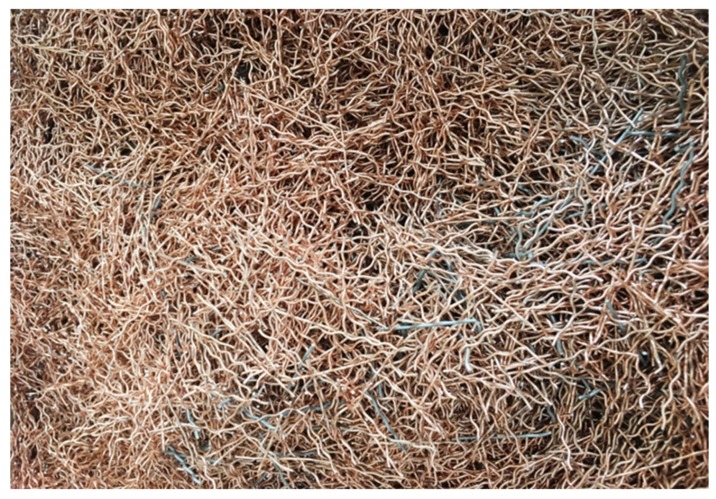
Copper-coated, crimped steel fibres.

Crimped, copper-coated steel fibres were chosen as fibre reinforcement (see Figure 6). This kind of steel fibre is one of the most popular in civil engineering [17,18], and was successfully used to reinforce concretes solely based on red ceramic waste [19]. The fibres proved to be compatible with such concretes in terms of properties of both fresh mixes and hardened composites. The geometric and mechanical properties of the used crimped steel fibres are summarised in Table 2.

## 3. Experimental Design

An ordinary integral simplex design (also known as a mixture design) [21] was utilised in this research. The three types of aggregate were named as follows: *X*—red ceramic waste; *Y*—natural aggregate; *Z*—white ceramic waste. Due to the different specific gravity values of red ceramic waste, white ceramic waste, and natural aggregate, the materials were dosed by volume. The specific property of the mixture design was that the sum of the volume of all three ingredients was always equal to 100%. In this case, the three aggregates played the roles of the ingredients. The utilised design is described in detail in Table 3. 

The object of the experiment was considered to be a complex composite material. The internal structure of the material was unavailable (for unknown reasons) to observers, with only the “input” and “output” parameters known to observers [22,23]. All achieved experimental results were statistically processed. The Smirnov–Grubbs criterion was used to assess gross error of the values. The sequence of specific test realisations was decided using a digital random number generator to guarantee objectivity. All calculations associated with the execution of the research and graphic interpretation of the mathematical model were carried out using the Statistica 12 software suite. Contour plots were created using a polynomial fit with fitted functions characterised by a correlation coefficient of at least 0.80. This type of experimental design was successfully used numerous times in concrete technology, including concretes based on waste aggregates and steel fibre-reinforced concretes [21]. The number, shape, and size of specimens utilised for each test are presented in Table 4. 

All specimens were tested after 28 days of curing (first day in a plastic mould covered by a polyethylene sheet, then 27 days in a water tank) in a temperature of 20 °C ± 0.5 °C. After curing, specimens were measured, weighed, and dried to avoid problems during the ultrasound velocity test [25]. The calculated density was a general quality test of the prepared specimens, with the value of density also useful for the ultrasound propagation velocity test and for computing the dynamic modulus of elasticity value. The shear strength test was performed on half of the beams that remained after the flexural test. Concrete mixes were modified by adding steel fibres to proportions of 0.5%, 1%, and 1.5%. The achieved results were subsequently compared with results obtained by other researchers working on steel fibre-reinforced concrete. Altogether, 16 concrete mixes were cast in order to test the properties of steel fibre-reinforced concrete (SFRC) in the hardened state.

## 4. Results

**Figure 7:**
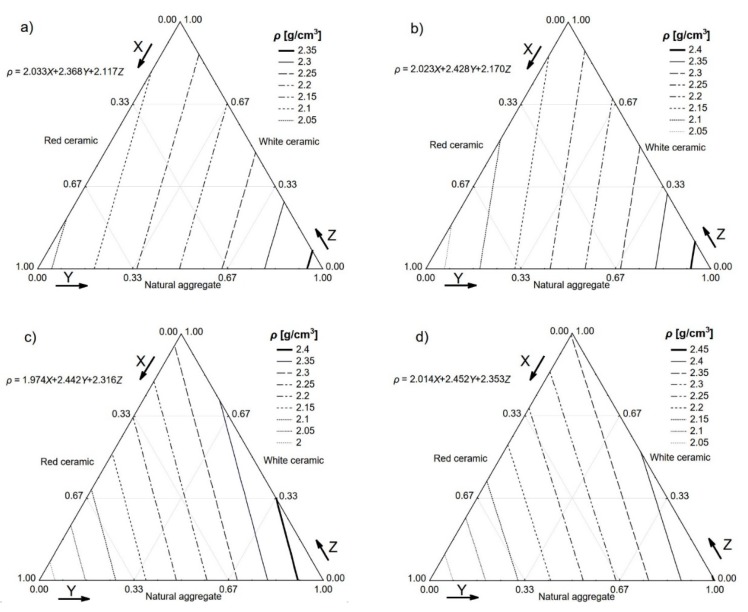
Densities (*ρ*) of the tested concretes. *V_f_*: (**a**) 0%; (**b**) 0.5%; (**c**) 1.0%; (**d**) 1.5%.

The densities of the hardened composites comprised the first property to be tested. The density of the mixes with no fibre ranged from 2.033 kg/dm^3^ for composites based solely on the red ceramic waste aggregate to values over 2.350 kg/dm^3^ for composites based solely on the natural aggregate. The composite created using only the white ceramic waste aggregate was characterised by a density of 2.117 kg/dm^3^. These results are presented in Figure 7a in the form of a ternary contour plot, where the *X*-axis represents the proportion of red ceramic in the concrete aggregate mix, the *Y*-axis represents the natural aggregate proportion, and the *Z*-axis represents the white ceramic proportion. Density graph lines can be seen within the triangle, representing the fractions of the aggregates. The densities of all the concrete mixes with the addition of steel fibre (see Figure 7b–d) were quite similar. Adding the steel fibre, which was characterised by a density of 7.600 kg/dm^3^, to the concrete mix, which was characterised by a density of roughly 2.200 kg/dm^3^, would increase its density. This phenomenon was described in the literature and is associated with extra air being trapped in mixes containing the fibre [26].

**Figure 8:**
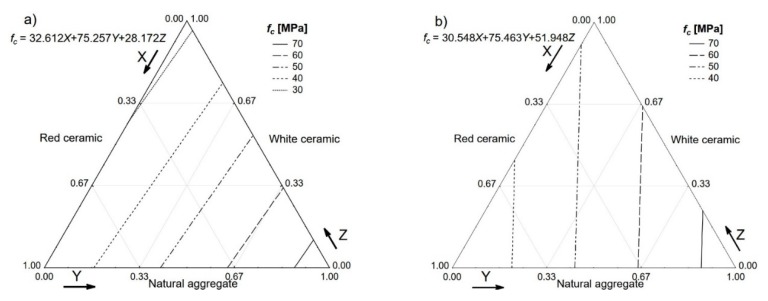

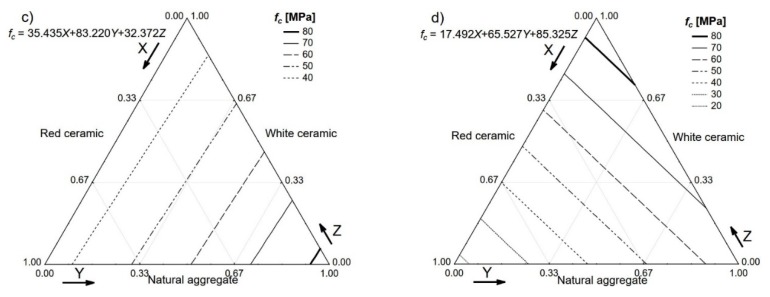
Compressive strength (*f_c_*) of the tested concretes. *V_f_*: (**a**) 0%; (**b**) 0.5%; (**c**) 1.0%; (**d**) 1.5%.

Results regarding the compressive strength tested on the cube specimens are presented in Figure 8. In the case of the SFRC based on an ordinary aggregate, the compressive strength is usually influenced by the addition of steel fibre in a very limited way [27]. A very different phenomenon is presented in Figure 8.

The compressive strength ranged from 17.5 to 85.3 MPa for the SFRC with 1.5% steel fibre, which was solely based on red and white ceramic, respectively. The differences resulting from less steel fibre addition and the concrete mixes containing no fibre were smaller, but still significant.

Before destructive testing, all specimens were used to measure the ultrasonic pulse velocity (*V*). The velocity was influenced by the aggregate (red ceramic is porous, whereas white ceramic is nonporous) and the addition of steel fibre. The highest velocity was associated with no-fibre, white ceramic concrete (just over 5 km/s) and the lowest velocity was observed with the no-fibre, red ceramic concrete (almost 2.5 km/s). In theory, the addition of steel fibre should increase the ultrasonic pulse velocity, but the extra entrapped air effectively contradicted this process. All of the tested SFRCs were characterised by an ultrasonic pulse velocity ranging between the values achieved by the concretes with no fibre. Once the velocities were determined for all of the concretes in question, general ideas about their quality and uniformity could be attained. The velocity of the ultrasonic pulse was used to classify the quality of the tested concretes [28,29] using three main ranges, i.e., *V* > 4.5 km/s, 4.5 km/s > *V* > 3.5 km/s, and 3.5 km/s > *V* > 3.0 km/s, of velocity which was associated with concrete quality (excellent, good, or medium, respectively). Results below 3.0 km/s were recognised as unsatisfactory. Tested concretes covering the whole range of possible results demonstrated the significant impact of aggregates and fibre addition on the properties and qualities of cement composites. 

The value of the dynamic modulus of elasticity (*E_d_*) was then calculated, as described by Neville [24]. The calculation was based on the assumption that the tested concrete was homogeneous, isotropic, and elastic. A simplified equation which did not require the value of Poisson’s ratio was used (see Equation (1)) [24].

*E_d_* = *ρ* · *V*^2^ (1)

where *ρ* is the density of hardened concrete (kg/m^3^) and *V* is the ultrasonic pulse velocity (m/s).

**Figure 9:**
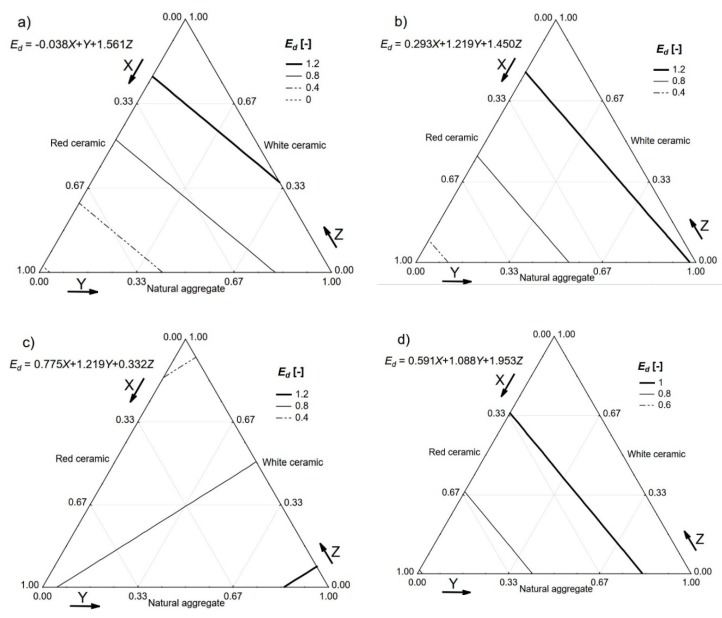
Dynamic modulus of elasticity *E_d_* (relative scale) of the tested concretes. *V_f_*: (**a**) 0%; (**b**) 0.5%; (**c**) 1.0%; (**d**) 1.5%.

Cement composites do not fulfil the physical requirements for the validity of the above equation, nevertheless, numerous researchers [28,29,30] successfully utilised it for quality control and technical checks of concrete structures in service. The test is very handy for the detection of voids, deterioration processes of different origins, and general uniformity of the cast concrete [31]. The dynamic modulus of elasticity values calculated for all of the tested concretes is presented in Figure 9 with the help of a relative scale.

**Figure 10:**
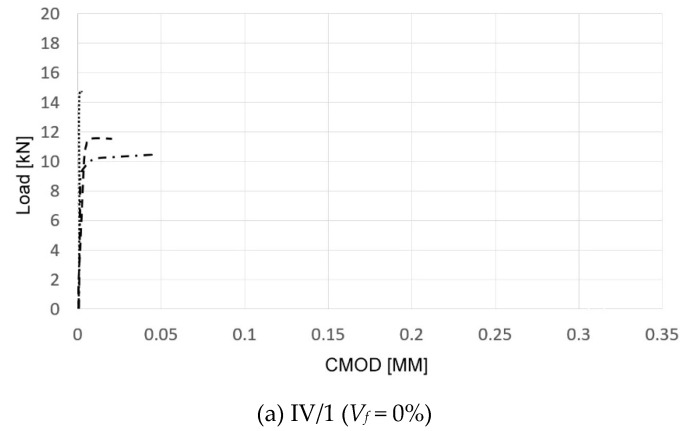

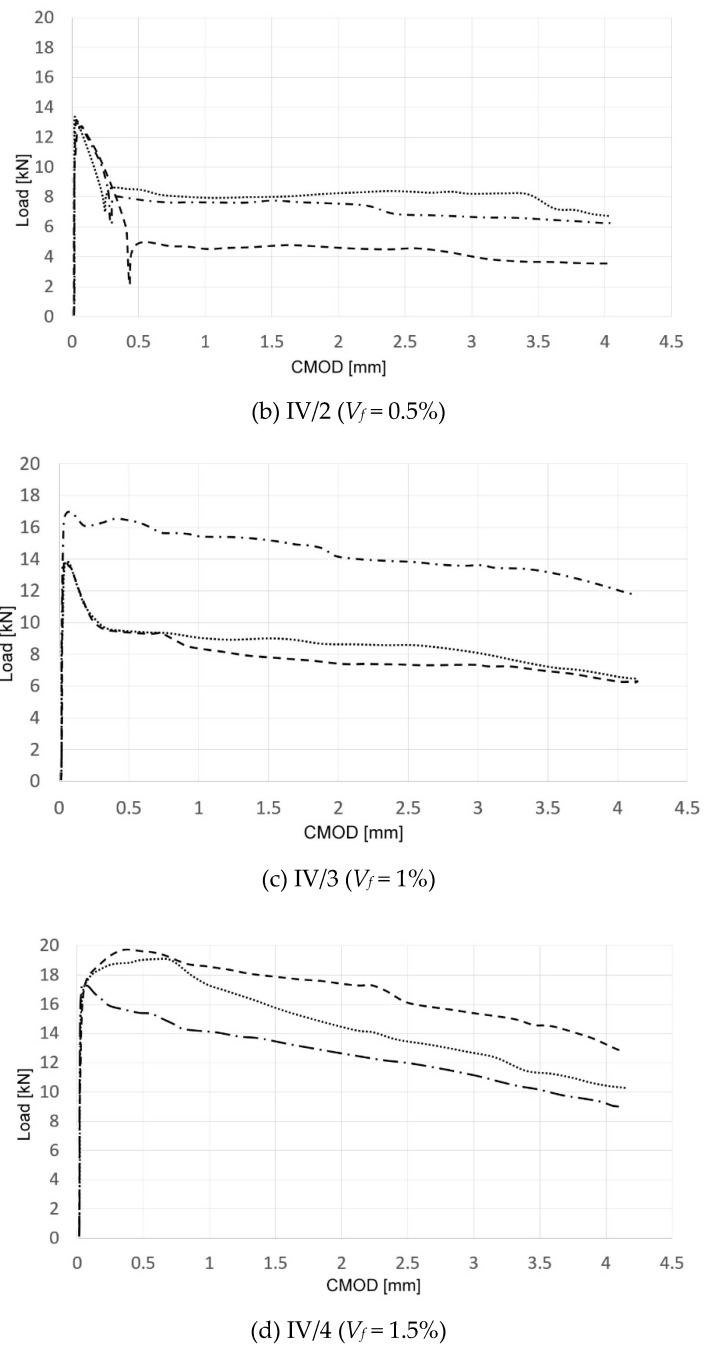
Load–crack mouth opening displacement (CMOD) diagrams (three specimens per SFRC).

Assessment of flexural characteristics of the concretes in question was based on the registered load–crack mouth opening displacement (CMOD, as defined in EN 14651:2007) relationship. Specimens were considered to be ultimately destroyed after achieving a CMOD equal to 4 mm. Exemplary registered load–CMOD diagrams are presented in Figure 10.

**Figure 11:**
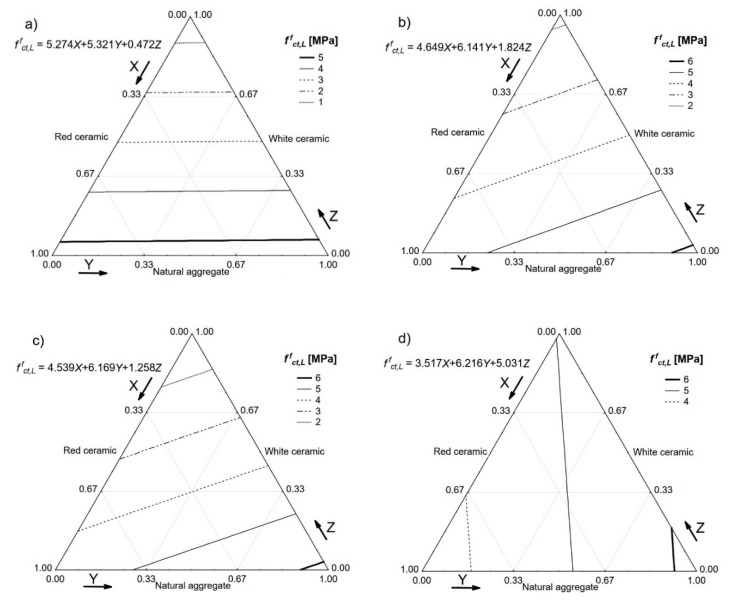
Limit of proportionality *f_LOP_* of the tested concretes. *V_f_*: (**a**) 0%; (**b**) 0.5%; (**c**) 1.0%; (**d**) 1.5%.

The load–CMOD diagrams were used to define the limit of proportionality *f_LOP_* of a particular concrete and all four residual strengths, i.e., *f_R1_, f_R2_, f_R3_*, and *f_R4_.* The achieved values of *f_LOP_* are presented in Figure 11.

**Figure 12:**
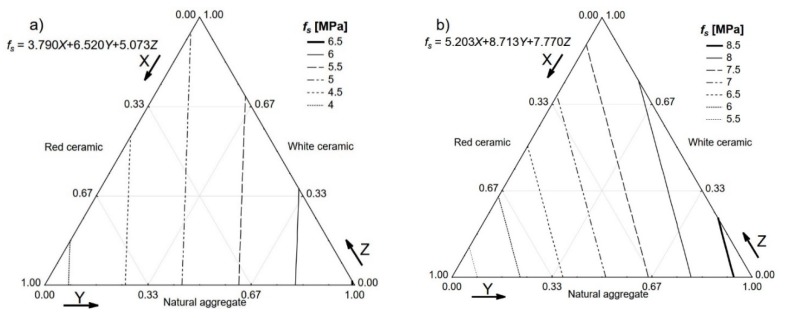

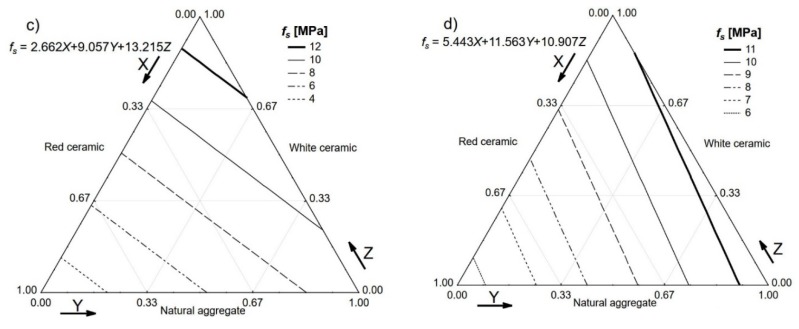
Shear strength *f_s_* of the tested concretes. *V_f_*: (**a**) 0%; (**b**) 0.5%; (**c**) 1.0%; (**d**) 1.5%.

The last tested property was shear strength, which is very important in the case of SFRC because it differentiates these materials significantly from ordinary concretes. The achieved results of shear strength are presented in Figure 12. SFRCs with high volumes of fibre were characterised by almost twice the shear strength of that exhibited by concrete-based aggregates with no fibre.

## 5. Discussion

Four residual strengths (*f_R1_*, *f_R2_*, *f_R3_*, *f_R4_*) associated with particular CMOD values (0.5, 1.5, 2.5, and 3.5 mm) are not feasible for the direct design of an SFRC mix. There is general agreement among the global SFRC research community that the first residual strength *f_R1_* is important in terms of service conditions, whereas the third residual strength *f_R3_* is recognised as a key factor for the assessment of ultimate conditions. The “*fib* Bulletin 55, Model Code 2010” proposed the utilisation of the first and third residual strengths to calculate both the serviceability limit state (SLS) and the ultimate limit state (USL). Basically, the ratio *f_R3_*/*f_R1_* was defined to describe the relationship between the behaviour of SFRC at ULS and SLS [32,33].

The proposed *fib* strength classification of SFRC consisted of two elements, i.e., strength interval (namely *f_R1_*) and post-cracking softening or hardening behaviour, which was described by a letter symbol from *a* to *d* directly referring to the *f_R3_*/*f_R1_* ratio. Letter *a* represents the strongest softening and letter *d* represents the strongest hardening [34].

When the fibre reinforcement was efficient enough, substitution of traditional bar and stirrup reinforcement was enabled. Two following conditions were fulfilled simultaneously to pass the substitution threshold:

*f_R1_* / *f_LOP_* > 0.4 (2)

*f_R3_* / *f_R1_* > 0.5 (3)

Calculated values of the above factors for tested concretes are presented in Table 5 with the associated strength class and reinforcement substitution. 

The achieved strength classes and enabled traditional reinforcement substitutions allowed for the utilisation of the tested concretes for structural applications. The wise use of different blends of ceramic waste and natural aggregates to shape the properties of cast concretes is possible. The proposed approach toward ceramic waste aggregates merged the advantages of internal curing and fibre reinforcement and was proven to be efficient.

## 6. Conclusions

The following conclusions can be drawn from the research described in this paper:

- It is possible to cast composites based on multiple waste aggregates;
- A blend of waste ceramic aggregates achieved a greater flexural strength of a cement composite than ordinary natural sand;
- The highest compressive strength was achieved using only natural aggregates;
- The compressive strength of the tested concretes was significantly influenced by the composition of the aggregate mix, as evidenced by the concrete with 1.5% fibre composition, whereby the values ranged from 17.5 MPa to 85.3 MPa;
- It is possible to partially or fully substitute natural aggregates with white or red ceramic wastes;
- The composites created on the basis of the white and red ceramic wastes are characterised by satisfactory mechanical properties, allowing for the assignment of standard strength classes according to both the EN and *fib* Model Code 2010;
- The research programme should be continued using greater specimens, focusing on more complicated mechanical characteristics (e.g., dynamic properties) of composites to enable full-scale modelling.

## Figures and Tables

**Table 1:** Granulometric and other properties of the used aggregates.

Sieves (mm)	Passing (%)		
	Natural	Red Ceramic	White Ceramic
0.000–0.125	1.0	7.0	3.0
0.125–0.250	6.0	17.0	10.0
0.250–0.500	18.5	25.5	16.0
0.500–1.000	27.0	32.0	25.0
1.000–2.000	36.0	40.0	39.0
2.000–4.000	55.0	52.0	55.5
4.000–8.000	77.0	70.0	77.0
8.000–16.00	100.0	100.0	100.0
*d_m_* (mm)	3.47	3.67	3.33
*m_K_* (-)	5.795	5.565	5.744
*m_H_* (-)	141.850	134.950	140.323
*m_A_* (-)	4.715	4.485	4.664
*ρ* (kg/m^3^)	2650	1690	2400
Absorbability (%)	-	16.8	0.0
Freeze–thaw resistance (%) of mass loss	0.06	0.17	0.01
Abrasion resistance (%) of LA mass loss	24	44	24.5

**Table 2:** Properties of copper-coated steel fibres [19].

Dimensions (mm)		*FIER* *	*l*/*d*	Density	Ultimate Tensile Strength
Length (*l*)	Diameter (*d*)	(-)	(-)	(kg/m^3^)	(GPa)
30.8	0.73	169.4	42.3	7800	1.7 ± 0.3

* Fibre intrinsic efficiency ratio [20].

**Table 3:** Aggregate mixes.

Mix No.	Aggregate (%)		
	Natural Aggregate	White Ceramic Waste	Red Ceramic Waste
I	100	0	0
II	0	33	67
III	67	33	0
IV	34	33	33

**Table 4:** Number of specimens, shapes, dimensions, and testing procedures.

Type of Test	Specimen Shape (cm)	Number of Specimens		Standard
		In One Test	Total	
Density	Cube 15 × 15 × 15 Beam 70 × 15 × 15	3 3	96	EN 12390-7:2011 *
Compression strength	Cube 15 × 15 × 15	3	48	EN 12390-3:2011 *
Shear strength	Beam 70 × 15 × 15	3	48	JCI-SF6:1984 **
Ultrasound propagation velocity	Beam 70 × 15 × 15	3	48	EN 12504-4:2005 *
Dynamic modulus of elasticity ***	Beam 70 × 15 × 15	3	48	EN 12504-4:2005 *
Flexural strength: LOP (limit of proportionality)	Beam 70 × 15 × 15	3	48	EN 14651:2007 *

* European standard; ** Japanese standard; *** as defined by Neville [24].

**Table 5:** Strength classes of tested steel fibre-reinforced concrete (SFRC) with *V_f_* = 1.5% according to “*fib* Bulletin 55, Model Code 2010”.

Concrete Symbol	*f_R3_/f_R1_*	*f_R1_/f_LOP_*	*f_LOP_* (MPa)	Strength Class	Reinforcement Substitution
I	0.783	0.978	6.216	6.0b	Enabled
II	0.696	0.597	3.653	3.0a	Enabled
III	0.788	1.005	5.452	5.0b	Enabled
IV	0.761	1.027	5.658	5.0b	Enabled
